# Tea

**Published:** 1875-04

**Authors:** 


Nearly the whole of the black tea annually brought to this country belongs to the class called Congou, or "labor" tea. The leaves are exposed to the sun, or in an airy place. The object of this is not to dry them, but only to wilt them slowly and thoroughly. A quantity of the leaves, thus wilted, is put into a vessel, usually made of the splints of the bamboo, and trodden down together for a considerable time, until all the fibres and stems of the leaves are broken. Men, barefooted, are employed to do this work, because the Chinese do not appear to have found out a more convenient, expeditious, and effective method of attaining the object in view.

The leaves are then rolled, in a peculiar manner, by the hands of the operator, the object being to cause them to take a round or spiral form. If not rolled in this way, they would remain flat, a shape not adapted to the foreign market. While lying on the vessel, the hands, spread out, are passed around for some time, in a circular manner, parallel to the bottom of the vessel, lightly touching the leaves. The leaves are then placed in a heap, to heat for half an hour or longer, until they become of a reddish appearance. They are then spread out in the sun, or in a light and airy place, and then left to dry. The leaf is next sold to the agents of foreigners, or to native dealers, who take it away, and expend a great deal of labor upon it before it is shipped to foreign countries. It is sifted in coarse sieves, and picked over several times, in order to separate the different qualities, to remove the stems, the large or flat leaves, &c. It is dried several times over slow fires in iron pans, in order to prevent its spoiling through any moisture that may still be retained in it. It gets a great deal of handling and considerable dirt.

The process necessary to make Oolong is very simple; in fact, such tea is the pure article, in its most unsophisticated form, and with the least amount of manipulation. The green leaves are plucked from the bushes and gathered into baskets by women and children; they are then spread on a covered floor for twenty-four hours; then stirred and tossed in a metal pan over the fire, until they attain a curled-up and spongy appearance, and possess the proper smell. Finally they are *fired* in a wicker basket, shaped like an egg-cup, the waist of which is divided by a sieve, upon which about seven pounds of tea are placed; the basket is set over an open charcoal oven, the fire of which has been previously banked up with lime and ashes, and emits no smoke. The Oolong, however, when sold to the foreigner, has not been sufficiently fired to withstand the trying effect of a long voyage home, and has to re-undergo the latter process for six or eight hours, before it is finally packed into chests for export.

The Chinese themselves use the simple decoction of tea without any addition of sugar or milk, and pour off the infusion almost directly after the boiling water has been poured on the leaf. They also frequently make their tea in a cup provided with a cover. There are other plants used for tea by the poorer Chinese; the leaves of one or two species of camelia are sometimes employed for the purpose, in districts where they are abundant; but these and other plants are considered poor substitutes for the true tea by the natives themselves.

